# ‘It's a job to be done’. Managing polypharmacy at home: A qualitative interview study exploring the experiences of older people living with frailty

**DOI:** 10.1111/hex.13952

**Published:** 2024-01-10

**Authors:** Giorgia Previdoli, David P. Alldred, Jonathan Silcock, Savi Tyndale‐Biscoe, Daniel Okeowo, V‐Lin Cheong, Beth Fylan

**Affiliations:** ^1^ School of Pharmacy and Medical Sciences, Faculty of Life Sciences University of Bradford Bradford UK; ^2^ NIHR Yorkshire and Humber Patient Safety Research Collaboration Bradford UK; ^3^ Department of Health Sciences University of York York UK; ^4^ School of Healthcare, Faculty of Medicine and Health University of Leeds Leeds UK; ^5^ Bradford Teaching Hospitals NHS Foundation Trust Bradford UK; ^6^ School of Pharmacy Newcastle University Newcastle upon Tyne UK; ^7^ Leeds Teaching Hospitals NHS Trust Leeds UK

**Keywords:** frailty, medications management, medications self‐management, older people, polypharmacy, qualitative study

## Abstract

**Introduction:**

Many older people live with both multiple long‐term conditions and frailty; thus, they manage complex medicines regimens and are at heightened risk of the consequences of medicines errors. Research to enhance how people manage medicines has focused on adherence to regimens rather than on the wider skills necessary to safely manage medicines, and the older population living with frailty and managing multiple medicines at home has been under‐explored. This study, therefore, examines in depth how older people with mild to moderate frailty manage their polypharmacy regimens at home.

**Methods:**

Between June 2021 and February 2022, 32 patients aged 65 years or older with mild or moderate frailty and taking five or more medicines were recruited from 10 medical practices in the North of England, United Kingdom, and the CARE 75+ research cohort. Semi‐structured interviews were conducted face to face, by telephone or online. The interviews were recorded, transcribed verbatim and analysed using reflexive thematic analysis.

**Findings:**

Five themes were developed: (1) Managing many medicines is a skilled job I didn't apply for; (2) Medicines keep me going, but what happened to my life?; (3) Managing medicines in an unclear system; (4) Support with medicines that makes my work easier; and (5) My medicines are familiar to me—there is nothing else I need (or want) to know.

While navigating fragmented care, patients were expected to fit new medicines routines into their lives and keep on top of their medicines supply. Sometimes, they felt let down by a system that created new obstacles instead of supporting their complex daily work.

**Conclusion:**

Frail older patients, who are at heightened risk of the impact of medicines errors, are expected to perform complex work to safely self‐manage multiple medicines at home. Such a workload needs to be acknowledged, and more needs to be done to prepare people in order to avoid harm from medicines.

**Patient and Public Involvement:**

An older person managing multiple medicines at home was a core member of the research team. An advisory group of older patients and family members advised the study and was involved in the first stages of data analysis. This influenced how data were coded and themes shaped.

## 1 INTRODUCTION

Unsafe medication practices and medication errors are a leading cause of avoidable harm around the world.^1^ To address this issue, the World Health Organization launched a Global Patient Safety Challenge in 2017,^2^ aiming to reduce preventable harm from medicines by 50%, and renewed the call in 2022,^3^ identifying polypharmacy (taking five or more medicines) as a key area for improvement.^4^

As people get older, they are more likely to develop multiple health conditions.^5^ While improved treatments can support effective management of chronic conditions such as hypertension, diabetes and cardiovascular disease,^6^ taking multiple medicines and managing complex medicines regimens can increase the risk of harm.^7^ Polypharmacy and the complexity of medicines regimens have been linked to negative health outcomes in older people including adverse drug events, an impact on physical and cognitive function, hospitalisation and mortality.^8^, ^9^, ^10^

Frailty is an ageing‐related process in which multiple body systems gradually lose their in‐built reserves, which makes it harder for people to bounce back from illness and stressors.^11^ Frailty affects around 10% of people aged 65 years or older and between 25% and 50% of those aged over 85 years. Since 2018, NHS England has adopted a population‐based stratification approach to systematically identify people, aged 65 and over, who are living with moderate and severe frailty,^12^ to target support and early intervention, mostly adopting the electronic frailty index (eFI).^13^

Adverse drug events and medicines interactions are more frequent in the frail older population and, when problems with medicines arise, frailty contributes to an increased risk of negative health outcomes,^14^ such as hospital admission or readmission.^15^ Numerous studies have explored patients' experiences of polypharmacy^16^, ^17^ but not specifically people with both polypharmacy and frailty. In addition, interventions to support self‐management of medicines have overlooked the frail older population.^18^, ^19^

A minority of studies has examined occurrences of medicines safety issues in people's homes,^20^ with some identifying polypharmacy and complexity of treatment as contributing factors in older adults.^21^ Previous studies, informed by a resilient healthcare (Safety II) approach,^22^ which highlights how variability plays an important contributory role in safety in complex systems,^23^ offered insight into the medicines management experiences of patients at discharge from hospital. Some older patients and their informal carers were found to play an important safety role, for example, in anticipating discrepancies and mitigating the occurrence of errors by facilitating communication between care settings.^24^ This study builds on this work, using resilient healthcare theory, and fills a gap in the literature by exploring the experiences of medicines self‐management of an under‐researched population (patients with mild to moderate frailty and polypharmacy) in an under‐researched context (day‐to‐day medicines self‐management at home) to focus on the broad range of activities involved. Resilient healthcare theory was used to bridge the disconnection between work around medicines ‘as imagined’,^22^ (e.g., by prescribers), and work around medicines ‘as it happens’ (by patients). This study was the first stage of a research project aimed at codesigning ways to support older people to safely self‐manage their multiple medicines at home. It aims to understand the experiences of medicines self‐management of frail older patients with polypharmacy living at home and the strategies that they adopt to bolster resilience in the medicines management system.

## 2 METHODS

### 2.1 Study design

A qualitative study was conducted in South and West Yorkshire, UK, between April 2021 and August 2022. A sample was determined of 32 participants from eight medical general practices to yield sufficient data to explore experiences of receiving healthcare from different organisations. Eligibility criteria included older age (65+), polypharmacy (five medicines or more) and mild to moderate frailty (eFI index score between 0.13 and 0.36) or offering unpaid medicines management support to a patient matching the inclusion criteria. Patients recently discharged from hospital (in the previous 4 weeks) were not included in order to minimise additional management burden and to enable our study to focus on the routine management of medicines at home.

Patients with moderate to advanced cognitive impairment and/or a diagnosis of dementia were not included in this study because their medicines management needs warrant separate investigation and are being explored in an additional study.

The full list of inclusion and exclusion criteria is reported in Table 1.

**Table 1:** Eligibility criteria.

To be eligible, participants needed to:	Participants were not included if:
Be aged 65 and over;	They had more advance frailty, advanced dementia or cognitive impairment;
Have mild or moderate frailty identified through their electronic frailty index score;	They had been discharged from hospital in the previous 4 weeks.
Use or been prescribed five or more medicines;	
Live at home;	
Manage their own medicines with or without informal support; or	
Offering unpaid medicines management support to a patient matching these criteria.	

To overcome recruitment challenges during the COVID‐19 pandemic, the number of recruiting sites was increased to 10, and an additional route for recruitment was introduced via the CARE 75+ database.^25^ CARE 75+ is a cohort of patients aged 75 and older, who are part of a national study on ageing and frailty. Study participants were, therefore, identified and screened in two ways: (a) a database search in the healthcare records of participating medical practices, followed by screening using eligibility criteria by practice staff; (b) a database search in the CARE75+ records (only patients interested in new research), followed by screening using the eligibility criteria by the research team. Eligible patients received an invitation letter either from their medical practice or the research team (CARE75+ cohort). If interested, they contacted the lead researcher on the study (Giorgia Previdoli) to learn more.

### 2.2 Consent

Patients received written information about the study, followed by an introductory phone call in which the researcher explained the content in the information sheet and answered any arising questions. If the person was happy to go ahead, an interview was arranged at their convenience, after receiving written consent or recording verbal consent. Interviews took place face to face at the patient's home or medical practice, or in a community venue of their choice. Alternatively, interviews could be conducted online using Zoom®, or via telephone. Information about age and ethnicity was collected before starting the interview. Participants were asked how many medicines they took, to ensure that they still met the inclusion criteria. Information on formulation was not collected, but all participants were reminded to include all types of medicines formulations in the total.

### 2.3 Data collection

An interview guide (Appendix S1), informed by a resilient healthcare framework,^26^ was developed in collaboration with a public contributor (Savi Tyndale‐Biscoe). Questions explored how participants learned about their medicines and their conditions, how they monitored their medicines, anticipated issues (e.g., with supply) and how they responded to problems and concerns. Interviews were conducted by Giorgia Previdoli, lasted between 30 and 80 minutes, were audio‐ or video‐recorded and transcribed verbatim.

### 2.4 Data analysis

Analysis was conducted through a constructivist paradigm where multiple realities can coexist and subjective knowledge is created through social interactions.^27^ All data were analysed inductively, using reflexive thematic analysis,^28^, ^29^ chosen as a method consistent with the paradigm. The flexible and iterative nature of processes was well matched to the exploratory nature of the research. Giorgia Previdoli led and conducted the analysis, in collaboration with team members, including social researchers Beth Fylan, Catherine Powell and George Peat; pharmacists David Phillip Alldred, Jonathan Silcock, V‐Lin Cheong and Daniel Okeowo; a patient with lived experience (Savi Tyndale‐Biscoe); and a group including both older patients and family members supporting older people with their medicines (R. D., J. S., P. E., S. B. and K. M.—Patient Advisory Group).

The six steps for reflexive thematic analysis described by Braun and Clarke^29^ were recursively followed. For part of the data set (10 out of 32 interviews), data familiarisation (Step 1) was conducted as a team, with Giorgia Previdoli as a facilitator. Involving multiple researchers and members of the public in the analysis was not intended to increase reliability of the coding;^28^, ^30^ instead, it was intended to enable Giorgia Previdoli to gain a more nuanced interpretation by being ‘sensitised’ to what healthcare professionals, patients and the family members found resonated with their experiences. A reflexive account of how multiple researchers, patients and family members were involved and contributed to the analysis is reported in Appendix S2. Familiarisation and coding for the full data set (Step 2) were conducted by Giorgia Previdoli, who periodically discussed ideas for codes and potential themes with Beth Fylan, as they were generated (Step 3). Candidate themes were then checked back against the whole data set, iteratively recombined and codes were revised if needed (Step 4). Giorgia Previdoli and Beth Fylan worked together to construct, define and name the final themes (Step 5) and to write up a thematic narrative supported by the data (Step 6).

## 3 RESULTS

### 3.1 Participant characteristics

Forty people expressed interest in joining the study. Two people could not be included because they had moved out of the area; six people had to withdraw either for health or personal reasons. Thirty‐two older patients consented to participate and were interviewed between July 2021 and February 2022. Eleven interviews took place face to face, 11 were conducted by phone and 10 online. One participant identified as Pakistani British and one as South American British. Thirty patients identified as White British, 17 identified as male, 15 as female and their ages ranged from 65 to 86 years (mean age = 77). The number of medicines that they were taking at the time of the interview varied between 5 and 15. The eFI score was only used for screening and not shared with the researcher. Details about participants' characteristics are reported in Appendix S3.

### 3.2 Patients' experiences of medicines self‐management

**Figure 1:**
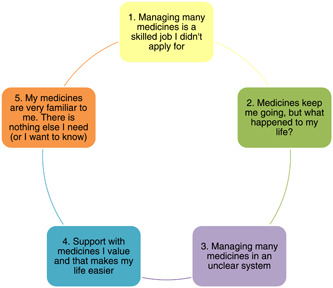
Themes developed using reflexive thematic analysis.

Five themes were developed, which are presented in Figure 1 and summarised below.

### 3.3 Theme 1: Managing many medicines is a skilled job I didn't apply for

This theme explores the complex and safety‐critical job of medicines self‐management, which demands time and dedication and requires multiple skills. Patients described adapting to frequent medicines changes, for example, increasing or decreasing doses, and fitting new medicines into their routines. Their strategies included always checking the medicines that they receive for mistakes, storing similar‐looking medicines separately and using prompts such as alarms to support timely administration. Complexity also arose from managing different medicines formulations, for example, tablets, injections, creams and eye drops, and needing to follow specific storage and dosing instructions for each one.

Amlodipine, aspirin, soluble aspirin, atorvastatin, ramipril, allopurinol, lansoprazole, also a nitro‐glycerine spray, CREON®. I also have got two hypodermic needles there for my insulin […] So I have got a blood sugar metre and I have got a 24‐hour insulin pen and I have a fast‐acting insulin pen. […] Er … also, they've put me on vitamin supplements because my weight went right down. (PP6)

Participants referred to the range of skills and knowledge that they needed, including understanding medicines and changes; monitoring their adherence and how they feel; anticipating problems; and concentrating to avoid mistakes, for example, when refilling compliance aids. They used online ordering systems, planned ahead to maintain supplies, detected errors and took action.

Well, I suppose you need a few spatial skills to make sure you put them [medicines] in the right [compartment of the compliance aid], I mean and you need to understand the ones that are morning, and the ones that are evening. And I think you need to concentrate as well. (PP12)

Well, yeah, because the packages that I'm taking the medicine out of are the same packaging. So if you take one tablet out of it and you only take two tablets a day, at the end of that day there should be two empty slots. If there are only one empty slot, you've not taken your tablet. (PP29)

Despite the challenges, some participants described feeling confident and content with their medicines and the way they were managing them. Being good at keeping on top of their multiple medicines, explained a few, made them feel in control and proud.

I like it [organising and managing my medicines], I really like doing it, you know, it makes me feel good that I'm capable and I can do it and I know why. (PP14)

### 3.4 Theme 2: Medicines keep me going, but what happened to my life?

This theme describes how patients tried to balance the demands of their medicines regimens with the desire to continue their normal lives. Some considered the benefits of taking medicines to be greater than the risks; however, the side effects that they attributed to their medicines (such as feeling in pain or ‘slowed down’) impacted their quality of life and the activities that they had previously enjoyed. Some autonomously adapted their regimens, for example, avoiding taking medicines at specific times, or stopped taking them altogether. Others expressed concern about taking too many medicines, becoming addicted or that they were continuing medicines that should have been prescribed for a limited period.

I do sometimes wish I weren't taking as many tablets, but while ever it's keeping me going, I'll take them [laughs] […] Is the side‐effects better than, you know, what you're taking them for? […] That sort of thing goes through me head sometimes. (PP4)

I made the decision to stop taking statin because I feel better. Living is more important … if you said to me, right you can have pain‐free now for the next year and a really nice time and then you will die, I would say, I'll take it. (PP1)

Many expressed how the work involved in organising and taking medicines had been absorbed into, and in some cases determined, their daily routines, for example, the time that they woke up, had their meals or went to bed. Some patients worried about forgetting doses and developed strategies to ensure their adherence. Those who managed family members' medicines in addition to their own reported a heightened burden.

You have to take it first thing in the morning, you have to stand up or at least sit upright, you can't eat or drink for half an hour after you've done it, half an hour to an hour, and you have to be careful. (PP2)

Before he passed away, we were taking tablets at eight different times during the day, and I must admit that did take a bit of organising … I was doing it, yes. Yes, he couldn't do it himself I was doing it for him. Even setting the alarm in the middle of the night! (PP10)

### 3.5 Theme 3: Managing many medicines in an unclear system

This theme explores how patients described their experiences of managing medicines in a fragmented care system, which was sometimes difficult to access and navigate. A few participants explained how, at times, they felt overlooked, unheard or misunderstood by healthcare professionals in relation to their medicines and how they relied on input from different poorly coordinated healthcare professionals, and they themselves needed to be proactive in spanning communication gaps.

I'm finding that difficult. It's between three lots, both consultants and the surgery and, yeah, and it's difficult for them because, you know, it's changing each time, and I phone the surgery and say, ‘I know my prescription needs to change because I was told that at the consultation’, and they say, ‘No, we haven't got a letter from them, we can't change it’. (PP31)

Patients explained that they had learned how to self‐manage medicines through trial and error, and some wished that their care team had more time to discuss risks and benefits of treatments or to consider alternative treatments. Finally, two patients described concern that nobody, apart from them, seemed to worry about the interactions between their different conditions and their multiple medicines. Many said that their medical practice would be their first point of contact for medicines‐related queries but talking to staff at their local practice had become difficult, particularly during the COVID‐19 pandemic. One patient, for example, decided to increase the dose of one medicine, following the instructions on the leaflet, while unable to reach the doctor. Some patients reported that they had not been offered a medicines review or they were not sure if their medicines had been reviewed or not.

[I haven't had a review] for the last two years, because you can't see a GP. You can't get to speak to anybody. (PP9)

### 3.6 Theme 4: the support with medicines I value and that makes my work easier

This theme describes practical and emotional support that people received from their own networks and healthcare professionals. Support from others included being reminded to take medicines, or mutual support from a partner, for example, in setting up shared reminders on phone calendars or support collecting medicines.

Well, we put alarms on the, on his phone, so that it goes off when we need to take medicines, but I don't really help him with his medication because he's still able to do it himself. He's very, very independent. (CC2 and PP23)

A few described how they felt reassured by the idea of having people around them, willing to help, while others expressed concern about the idea of becoming a burden. Some anticipated not being able to manage medicines as they aged and had started preparing their support network.

My son and daughter‐in‐law, they would definitely know what to do if I needed medication … They know exactly where I keep everything and what I do because I made them aware of that because of my age and the fact that I may not always be so capable. (PP14)

Some participants talked about established good relationships with local doctors and community pharmacy staff. Being recognised as individuals, they explained, felt valuable. Talking to the only doctor who had known her for years, explained one patient, was essential in finding solutions to multiple issues that other doctors seemed unable to fix. Some patients described responding to problems by contacting their doctors or community nurses. Community pharmacists and NHS 111 were contacted when they suspected errors, adverse events or if they were worried about missing doses.

So [the diabetes nurse] started me on some tablets and I just can't remember the name of them, but after two weeks I was very ill… I was being sick all the time. So I went back to her and said: ‘You know I've never been sick before til I started taking these tablets’ and she said: ‘It could be a side effect, stop taking them’, so I did. (PP19)

He [doctor] was brilliant and he really listened to what I was saying, and he would think about, analyse and discuss it with me what he thought.… So, he was fantastic but other the GPs (family doctors), I know it's the time, actually, it's tick, tick, tick, 10 minutes out you go. (PP20)

Participants gave many examples of how they were supported in managing medicines. This included pharmacies delivering their medicines, pharmacists dispensing their tablets in bottles, to help them overcome dexterity issues and practice staff sending text reminders when they were due tests. Participants also described appreciating professionals following up after a new medicine was introduced, offering alternatives if side effects were reported or reducing the number of times that they needed to take medicines during the day.

I had terrible trouble because if you take your tablets at 8 o'clock in the morning, at 8 o'clock at night you don't know where you're going to be anyway, and I'd be in bed and I'd think, oh, forgot my tablet […] So it was the chemist actually, who was going through them, and he said: ‘You can have one that you just take once a day’…. So, that makes that a lot easier for me, because it's just once a day. (PP30)

### 3.7 Theme 5: My medicines are very familiar to me. There is nothing else I need (or want) to know or worry about

This theme describes how some patients were familiar with their medicines after taking them for many years. For some, medicines were embedded in their daily routines, and they took them automatically, sometimes without knowing what they were. A few said that they never thought of carrying their own list of medicines (or most up‐to‐date prescription) to medical appointments, because they assumed that every healthcare professional, everywhere in the system, had access all the time to the most up‐to‐date information about their care, medicines included. Some patients reported that they would not question the decision made by a healthcare professional, nor felt the need to know more about their medicines. A few patients used words like ‘faith’, ‘acceptance’ and ‘getting on with it’ to describe their reaction to decisions made by their doctors. Others said that, because they trusted their healthcare team, they did not feel the need to check that the medicines received were correct.

I have no problem, it's a very simple operation. I've never questioned with my GP as to whether it should change, I'm in the hands of the professionals. (PP24)

## 4 DISCUSSION

This study explored the medicines management experiences of older people who live with mild and moderate frailty and who were taking five or more medicines. This research adds to the growing body of evidence around the complexity and multiple skills required to safely manage medicines at home. It adds a new perspective through the research cohort (older people living with frailty), demonstrating that despite their increased vulnerability to poor outcomes from medicines errors, the system places them at risk and places demands upon them to keep safe.

Patients described the activities required as a skilled job made more demanding by operating in a fragmented system. Participants expressed mixed feelings towards the impact that medicines had on their life, with some communicating the burden that they experienced. Most participants appreciated the practical help and encouragement received from their support networks. Many were proactive in their self‐management and sought discussions about treatment options with healthcare professionals. Others preferred to ‘do as they were told’ and avoided involvement, trusting in the healthcare system to perform optimally.

### 4.1 Workload of medicines management

Participants identified the numerous activities necessary to safely managing polypharmacy. These included keeping on top of supply; planning orders and collection; safely storing and disposing; monitoring effects and side effects; having a system in place to remember to take the right medicines at the right time; and coordinating inputs on treatments from different professionals. Our data add to the findings from recent observational studies about how older patients organise their work around medicines. Tasks performed by patients included ordering; organising and storing medicines;^31^, ^32^ planning the taking;^33^ monitoring and eventually reporting effects, reactions and interactions; and coordinating tests and appointments.^34^ Our data confirmed that safe medicines self‐management requires a wide range of knowledge and skills^18^, ^35^ If we acknowledge that medicines self‐management by patients with polypharmacy implies skilled work,^33^ we cannot ignore the inherent inequalities in, for example, health literacy, levels of deprivation, language barriers,^36^ race and ethnicity^37^ and access to social support.^38^ In the United Kingdom, the COVID‐19 pandemic brought to light how race and ethnicity affect people's access to and experiences of care.^39^ Research about how health inequalities impact on medicines self‐management experiences in the older people is still limited,^37^ and interventions to support frail older people on polypharmacy should take care not to widen health inequalities.^40^

### 4.2 Patient roles in the safety of medicines management

Participants in this study described the complexity of their medicines regimens and many were cognisant of threats to their safety. In response, they were vigilant, checking the supplies that they received, monitoring their symptoms and keeping track of their own adherence. Participants anticipated problems, like, for example, adverse effects from interacting medicines, and took action to prevent them. They also learnt from previous experiences, such as forgetting which medicines they were taking, and changed their behaviour, for example, by creating a list. Previous research has highlighted the under‐recognised role that patients play in medicines safety at a transition of care.^41^, ^42^ Here, we echo Lang et al.^43^ highlighting that their skills are crucial in maintaining safety in the day‐to‐day management of medicines, not solely at a time of heightened risk in their care, such as after hospital discharge. Our study shows that there are clear opportunities to formalise and support patient roles, including in this vulnerable population living with frailty, for example, by providing support tools and guidance and additional routes to report and resolve errors, empowering those who are able and willing to engage and exploring alternatives for those who cannot or prefer not to (e.g., involving their support network if appropriate or monitor more closely and frequently, if possible).

### 4.3 Burden of treatment

Many participants described reduced opportunities to enjoy life, either because of side effects or the demands posed by their complex regimens. This resonates with research into the burden of treatment^44^, ^45^ and highlights how the effort required to organise and take many medicines impacts on quality of life. Our data confirm that older patients with polypharmacy are particularly exposed to experiencing treatment burden^16^, ^44^, ^46^, ^47^ and problems related to medicines.^48^ Previous research described how treatment burden may remain undetected by healthcare professionals, especially in an overstretched healthcare system functioning in reactive mode.^46^ Patients in this study adopted their own strategies to reduce burden, such as skipping doses or withdrawing treatments. Due to difficulties in communicating with the healthcare team, in some cases, decisions were made without discussing options with healthcare professionals, with potentially serious safety implications. Structured medication reviews have played a central role in the attempt to mitigate problems with medicines and improve adherence in frail older people,^49^ along with the increasing number of deprescribing^50^ interventions aimed at the older population living with polypharmacy^51^ and frailty.^52^ Less work has explored how older patients could be better prepared and supported to self‐manage their medicines at home.^19^

Nevertheless, increased engagement in medicines management does not appeal to everyone. Some participants in this study were concerned with being overwhelmed by information about medicines and preferred taking their medicines without thinking, rather than engaging in demanding decision‐making processes. Further work is needed to explore what additional risks less activated or able patients living with frailty may face (e.g., inability to identify adverse events or medicines errors) in managing their medicines and what measures could mitigate them.

### 4.4 Managing medicines in a fragmented system

Fragmentation is acknowledged as a patient safety risk, especially at care transitions.^53^ Participants in this study, who had not recently experienced a transition of care, found system fragmentation a cause of frustration and an additional source of complexity. Some were able to anticipate problems due to poor communication across sectors. Some, for example, explained that they carried an updated list of medicines so that if they were admitted to hospital, staff would know what medicines they were taking, even if they had no access to primary care or pharmacist records. Other patients reported actively filling the gaps in communication, for example, by checking that changes in their medicines suggested by specialists were actioned everywhere in the system to avoid errors and delays. This supports the findings from research conducted in the United Kingdom exploring the role that patients may play in keeping the system safe.^41^, ^54^, ^55^ Pharmacists, as medicines experts, may be useful to bridge the gaps within a fragmented system to minimise medicines errors, helped in this task by their increased involvement in supporting the old population.^56^

### 4.5 Help with medicines

All patients in this study said they were managing their medicines alone, but many explained that they received some level of support from family members and friends. Participants' descriptions of how patients and their family members organised some of the medicines work together support research into the ‘relational work’ involved in medicines management.^57^

### 4.6 Strengths and limitations

This qualitative study was conducted with a sample of 32 patients receiving UK primary care, which allowed an in‐depth exploration of participants' varied experiences of medicines self‐management. Including a patient as a coauthor and involving older patients managing multiple medicines and their family members in data analysis sensitised the researchers to the patients' perspectives. This helped them to develop themes that resonated with patients' and families' lived experiences.^58^

Rules related to social distancing were still in place during fieldwork, and most medical practices in the United Kingdom significantly changed the way they operated during COVID.^59^, ^60^, ^61^ Research has shown that among the most affected by those changes, in terms of health outcomes, were those with long‐term conditions and multimorbidity.^62^ Patients' perceptions and experiences were possibly influenced by those changes. Difficulties in accessing doctors for queries or having a phone instead of face‐to‐face appointments may have influenced how participants in this study described their challenges in finding answers and help with their medicines.

The study did not set out to compare the experiences of managing polypharmacy of people living with frailty with other patients on multiple medicines, so we cannot know if their experiences were different. Nor did we attempt to assess the differences for people with different levels of frailty. We do now know, however, that this vulnerable population experiences the impact of poorly calibrated medicines management systems faced by other less vulnerable patients.

The main limitation of this study is the limited ethnic diversity of the sample and its representativeness of the Yorkshire and Humber population, where according to the most recent Census (2021), almost 19% of people identified as other than White British (8.9% of people identified as Asian, 2.1% as Black, 2.1% as mixed, 4.5% as White other and 1% as other).^63^ Only two participants in this study identified as being from a minoritised group.^64^ Discussions took place in the team about which aspects of the research hindered the participation of patients from different ethnic backgrounds. Further input came from conversations with Nadeem Khan, Giorgia Previdoli's mentor, in a programme aimed at increasing minoritised groups' participation in research. The main barriers identified were a combination of research design (specifically the role played by the medical practice in inviting participation) and the disproportionate impact that the COVID‐19 pandemic had on medical practices located in the most deprived areas.^65^ Most data in this qualitative study were collected during the second year of the COVID pandemic. In this study, the selection of sites was designed to reflect the characteristics of an older frail population in South and West Yorkshire, so that a variety of older patients could receive invitations to take part. During the research, practices located in deprived areas, where the most diverse population lives, reported increased pressures and reduced capacity to engage. Lessons were learned and shared with the wider research team. Mitigation measures were taken to balance participants' ethnic background composition in the following stages of the research, of which this interview study forms part. Recommendations were developed and became part of the equality, diversity and inclusion strategy that the wider team was developing at the time. Solutions implemented included internal auditing sessions where research proposals are scrutinised to ensure that neither the eligibility criteria nor the screening method made it harder for some parts of the population to take part.

## 5 CONCLUSION

Managing multiple medicines is complex and demanding for older people living with frailty, a population at heightened risk of the impact of poorly managed medicines. Patients' and families' work needs to be acknowledged and appreciated by healthcare staff. Support needs to be targeted to patients' circumstances and preferences, empowering patients willing to engage and play proactive roles and exploring alternative approaches when patients cannot or prefer not to engage.

## AUTHOR CONTRIBUTIONS

**Giorgia Previdoli**: Conceptualisation; investigation; writing—original draft; methodology; validation; visualisation; writing—review and editing; formal analysis; project administration. **David P. Alldred**: Conceptualisation; funding acquisition; writing ‐ original draft; writing—review and editing; methodology; formal analysis; validation. **Jonathan Silcock**: Conceptualisation; funding acquisition; writing—original draft; methodology; writing—review and editing; formal analysis. **Savi Tyndale‐Biscoe**: Conceptualisation; funding acquisition; writing—original draft; methodology; writing—review and editing; formal analysis. **Daniel Okeowo**: Conceptualisation; writing—original draft; methodology; writing—review and editing; formal analysis. **V‐Lin Cheong**: Conceptualisation; funding acquisition; writing—original draft; methodology; writing—review and editing; formal analysis. **Beth Fylan**: Conceptualisation; funding acquisition; writing—original draft; methodology; validation; visualisation; writing—review and editing; formal analysis; project administration; supervision.

## CONFLICT OF INTEREST STATEMENT

The authors declare no conflicts of interest.

## Supporting information

Supporting information.

Supporting information.

Supporting information.

## Data Availability

Research data are not shared for reasons associated with confidentiality and protection of human privacy.

## References

[1] Donaldson LJ , Kelley ET , Dhingra‐Kumar N , Kieny MP , Sheikh A . Medication without harm: WHO's third global patient safety challenge. Lancet. 2017;389(10080):1680‐1681. 10.1016/S0140-6736(17)31047-4 28463129

[2] World Health Organization . Medication without harm strategic framework. 2023. Accessed August 30, 2023. https://www.who.int/initiatives/medication-without-harm

[3] World Health Organization . WHO calls for urgent action by countries for achieving medication without harm. September 16, 2022. Accessed August 30, 2023. https://www.who.int/news/item/16-09-2022-who-calls-for-urgent-action-by-countries-for-achieving-medication-without-harm PMC9994493 36261195

[4] World Health Organization . Strategic framework. 2018. Accessed August 30, 2023 https://cdn.who.int/media/docs/default-source/patient-safety/strategic-framework-medication-without-harm86c06fafdf0b4294bd23ec9667dfb95d.pdf?sfvrsn=b5cb2d66_2

[5] Chowdhury SR , Chandra Das D , Sunna TC , Beyene J , Hossain A . Global and regional prevalence of multimorbidity in the adult population in community settings: a systematic review and meta‐analysis. EClinicalMedicine. 2023;57:101860. 10.1016/j.eclinm.2023.101860 36864977 PMC9971315

[6] Daunt R , Curtin D , O'Mahony D . Polypharmacy stewardship: a novel approach to tackle a major public health crisis. Lancet Healthy Longev. 2023;4(5):e228‐e235. 10.1016/S2666-7568(23)00036-3 37030320

[7] World Health Organization . Integrated Health Services, Medication without Harm. Medication Safety in Polypharmacy. 2019. Accessed August 30, 2023 https://www.who.int/publications/i/item/WHO-UHC-SDS-2019.11

[8] Wastesson JW , Morin L , Tan ECK , Johnell K . An update on the clinical consequences of polypharmacy in older adults: a narrative review. Expert Opin Drug Saf. 2018;17(12):1185‐1196. 10.1080/14740338.2018.1546841 30540223

[9] Zazzara MB , Palmer K , Vetrano DL , Carfì A , Onder G . Adverse drug reactions in older adults: a narrative review of the literature. Eur Geriatr Med. 2021;12(3):463‐473. 10.1007/s41999-021-00481-9 33738772 PMC8149349

[10] Wimmer BC , Bell JS , Fastbom J , Wiese MD , Johnell K . Medication regimen complexity and polypharmacy as factors associated with all‐cause mortality in older people: a population‐based cohort study. Ann Pharmacother. 2016;50(2):89‐95. 10.1177/1060028015621071 26681444 PMC4714103

[11] Clegg A , Young J , Iliffe S , Rikkert MO , Rockwood K . Frailty in elderly people. Lancet. 2013;381(9868):752‐762. 10.1016/S0140-6736(12)62167-9 23395245 PMC4098658

[12] Age UK . What is frailty? Age UK. July 21, 2020. Accessed November 3, 2023. https://www.ageuk.org.uk/our-impact/policy-research/frailty-in-older-people/understanding-frailty/

[13] England NHS . NHS England—identifying frailty. 2023. Accessed November 3, 2023. https://www.england.nhs.uk/ourwork/clinical-policy/older-people/frailty/frailty-risk-identification/

[14] Bonaga B , Sánchez‐Jurado PM , Martínez‐Reig M , et al. Frailty, polypharmacy, and health outcomes in older adults: the frailty and dependence in albacete study. J Am Med Dir Assoc. 2018;19(1):46‐52. 10.1016/j.jamda.2017.07.008 28899661

[15] Toh JJY , Zhang H , Soh YY , Zhang Z , Wu XV . Prevalence and health outcomes of polypharmacy and hyperpolypharmacy in older adults with frailty: a systematic review and meta‐analysis. Ageing Res Rev. 2023;83:101811. 10.1016/j.arr.2022.101811 36455791

[16] Eriksen CU , Kyriakidis S , Christensen LD , et al. Medication‐related experiences of patients with polypharmacy: a systematic review of qualitative studies. BMJ Open. 2020;10(9):e036158. 10.1136/bmjopen-2019-036158 PMC7477975 32895268

[17] Mikkelsen TH , Søndergaard J , Kjaer NK , et al. Handling polypharmacy—a qualitative study using focus group interviews with older patients, their relatives, and healthcare professionals. BMC Geriatr. 2023;23(1):477. 10.1186/s12877-023-04131-6 37553585 PMC10410867

[18] Howell EH , Senapati A , Hsich E , Gorodeski EZ . Medication self‐management skills and cognitive impairment in older adults hospitalized for heart failure: a cross‐sectional study. SAGE Open Med. 2017;5:2050312117700301. 10.1177/2050312117700301 28540048 PMC5433792

[19] Previdoli G , Cheong VL , Alldred D , et al. A rapid review of interventions to improve medicine self‐management for older people living at home. Health Expect. 2023;26(3):945‐988. 10.1111/hex.13729 36919190 PMC10154809

[20] Mira JJ , Lorenzo S , Guilabert M , Navarro I , Pérez‐Jover V . A systematic review of patient medication error on self‐administering medication at home. Expert Opin Drug Saf. 2015;14(6):815‐838. 10.1517/14740338.2015.1026326 25774444

[21] Aldila F , Walpola RL . Medicine self‐administration errors in the older adult population: a systematic review. Res Soc Adm Pharm. 2021;17(11):1877‐1886. 10.1016/j.sapharm.2021.03.008 33811011

[22] Hollnagel E , Wears RL , Braithwaite J . From safety‐I to safety‐II: a white paper Australia The resilient health care net. University of Southern Denmark, University of Florida, USA, and Macquarie University. 2015. http://www.qpsolutions.vn/cgi-bin/Document/Safety%20II%20WhitePaper.pdf

[23] Braithwaite J , Churruca K , Ellis LA , et al. Complexity Science in Healthcare: Aspirations, Approaches, Applications and Accomplishments. Macmillan Education Australia; 2017.

[24] Tomlinson J , Silcock J , Smith H , Karban K , Fylan B . Post‐discharge medicines management: the experiences, perceptions and roles of older people and their family carers. Health Expect. 2020;23(6):1603‐1613. 10.1111/hex.13145 33063445 PMC7752204

[25] Heaven A , Brown L , Young J , et al. Community ageing research 75+ study (CARE75+): an experimental ageing and frailty research cohort. BMJ Open. 2019;9(3):e026744. 10.1136/bmjopen-2018-026744 PMC6429944 30850418

[26] Hollnagel E . Epilogue: RAG–the resilience analysis grid. In: Erik H , Jean P , David DW , John W , eds. Resilience Engineering in Practice. Ashgate; 2011:275‐296.

[27] Brown MEL , Dueñas AN . A medical science educator's guide to selecting a research paradigm: building a basis for better research. Med Sci Educ. 2020;30(1):545‐553. 10.1007/s40670-019-00898-9 34457699 PMC8368685

[28] Braun V , Clarke V . Reflecting on reflexive thematic analysis. Qual Res Sport Exerc Health. 2019;11(4):589‐597. 10.1080/2159676X.2019.1628806

[29] Braun V , Clarke V . Using thematic analysis in psychology. Qual Res Psychol. 2006;3(2):77‐101. 10.1191/1478088706qp063oa

[30] Braun V , Clarke V . Is thematic analysis used well in health psychology? A critical review of published research, with recommendations for quality practice and reporting. Health Psychol Rev. 2023;19:1‐24. 10.1080/17437199.2022.2161594 36656762

[31] Carli Lorenzini G , Bell A , Olsson A . “You need to be healthy to be sick”: exploring older people's experiences with medication packaging at home. Age Ageing. 2022;51(3):afac050. 10.1093/ageing/afac050 35258519 PMC8903009

[32] Vandermause R , Neumiller JJ , Gates BJ , et al. Preserving self: medication‐taking practices and preferences of older adults with multiple chronic medical conditions. J Nurs Scholarsh. 2016;48(6):533‐542. 10.1111/jnu.12250 27802372

[33] Swinglehurst D , Fudge N . Organising polypharmacy: unpacking medicines, unpacking meanings—an ethnographic study. BMJ Open. 2021;11(8):e049218. 10.1136/bmjopen-2021-049218 PMC8395269 34446490

[34] Bell C , Appel CW , Frølich A , Prior A , Vedsted P . Improving health care for patients with multimorbidity: a mixed‐methods study to explore the feasibility and process of aligning scheduled outpatient appointments through collaboration between medical specialties. Int J Integr Care. 2022;22(1):17. 10.5334/ijic.6013 PMC8896239 35340347

[35] Maidment I , Lawson S , Wong G , et al. Towards an understanding of the burdens of medication management affecting older people: the MEMORABLE realist synthesis. BMC Geriatr. 2020;20(1):183. 10.1186/s12877-020-01568-x 32498672 PMC7272211

[36] Lyson HC , Sharma AE , Cherian R , et al. A qualitative analysis of outpatient medication use in community settings: observed safety vulnerabilities and recommendations for improved patient safety. J Patient Saf. 2021;17(4):e335‐e342. 10.1097/PTS.0000000000000590 30882615 PMC7060148

[37] Secchi A , Booth A , Maidment I , Sud D , Zaman H . Medication management in minority, Asian and Black ethnic older people in the United Kingdom: a mixed‐studies systematic review. J Clin Pharm Ther. 2022;47(9):1322‐1336. 10.1111/jcpt.13735 35844186

[38] Chaplin S . What can we learn from patients' experiences of polypharmacy? Prescriber. 2021;32(4):21‐22. 10.1002/psb.1909

[39] Ajayi (Sotubo) O . A perspective on health inequalities in BAME communities and how to improve access to primary care. Future Healthc J. 2021;8(1):36‐39. 10.7861/fhj.2020-0217 33791458 PMC8004339

[40] Wade C , Malhotra AM , McGuire P , Vincent C , Fowler A . Action on patient safety can reduce health inequalities. BMJ. 2022;376:e067090. 10.1136/bmj-2021-067090 35351684 PMC9731338

[41] Fylan B , Armitage G , Naylor D , Blenkinsopp A . A qualitative study of patient involvement in medicines management after hospital discharge: an under‐recognised source of systems resilience. BMJ Qual Safe. 2018;27(7):539‐546. 10.1136/bmjqs-2017-006813 29146681

[42] Fylan B , Marques I , Ismail H , et al. Gaps, traps, bridges and props: a mixed‐methods study of resilience in the medicines management system for patients with heart failure at hospital discharge. BMJ Open. 2019;9(2):e023440. 10.1136/bmjopen-2018-023440 PMC6377507 30782879

[43] Lang A , Macdonald M , Marck P , et al. Seniors managing multiple medications: using mixed methods to view the home care safety lens. BMC Health Serv Res. 2015;15:548. 10.1186/s12913-015-1193-5 26651331 PMC4677040

[44] Mohammed MA , Moles RJ , Chen TF . Medication‐related burden and patients' lived experience with medicine: a systematic review and metasynthesis of qualitative studies. BMJ Open. 2016;6(2):e010035. 10.1136/bmjopen-2015-010035 PMC4746464 26839015

[45] Mair FS , May CR . Thinking about the burden of treatment. BMJ. 2014;349:g6680. 10.1136/bmj.g6680 25385748

[46] Maidment I , Huckerby C , Shukla D . Medication management in older people—a hidden burden. Prescriber. 2020;31(11‐12):30‐33. 10.1002/psb.1881

[47] van Merode T , van de Ven K , van den Akker M . Patients with multimorbidity and their treatment burden in different daily life domains: a qualitative study in primary care in the Netherlands and Belgium. J Comorb. 2018;8(1):9‐15. 10.15256/joc.2018.8.119 29651408 PMC5885066

[48] Nicosia FM , Spar MJ , Stebbins M , et al. What is a medication‐related problem? A qualitative study of older adults and primary care clinicians. J Gen Intern Med. 2020;35(3):724‐731. 10.1007/s11606-019-05463-z 31677102 PMC7080912

[49] Verma A , Saha S , Jarl J , Conlon E , McGuinness B , Trépel D . An overview of systematic reviews and meta‐analyses on the effect of medication interventions targeting polypharmacy for frail older adults. J Clin Med. 2023;12(4):1379. 10.3390/jcm12041379 36835915 PMC9960328

[50] O'Donnell LK , Ibrahim K . Polypharmacy and deprescribing: challenging the old and embracing the new. BMC Geriatr. 2022;22(1):734. 10.1186/s12877-022-03408-6 36068485 PMC9450314

[51] Ulley J , Harrop D , Ali A , Alton S , Fowler Davis S . Deprescribing interventions and their impact on medication adherence in community‐dwelling older adults with polypharmacy: a systematic review. BMC Geriatr. 2019;19(1):15. 10.1186/s12877-019-1031-4 30658576 PMC6339421

[52] Ibrahim K , Cox NJ , Stevenson JM , Lim S , Fraser SDS , Roberts HC . A systematic review of the evidence for deprescribing interventions among older people living with frailty. BMC Geriatr. 2021;21(1):258. 10.1186/s12877-021-02208-8 33865310 PMC8052791

[53] Picton C , Wright H . Keeping Patients Safe when they Transfer between Care Providers—Getting the Medicines Right. Royal Pharmaceutical Society; 2012.

[54] O'Hara JK , Aase K , Waring J . Scaffolding our systems? Patients and families “reaching in” as a source of healthcare resilience. BMJ Qual Safe. 2019;28(1):3‐6. 10.1136/bmjqs-2018-008216 29764929

[55] O'Hara JK , Canfield C , Aase K . Patient and family perspectives in resilient healthcare studies: a question of morality or logic? Safe Sci. 2019;120:99‐106. 10.1016/j.ssci.2019.06.024

[56] Davies N , Kolodin V , Woodward A , et al. Models of care and the role of clinical pharmacists in UK primary care for older adults: a scoping review protocol. PLoS One. 2023;18(7):e0276471. 10.1371/journal.pone.0276471 37498969 PMC10374084

[57] Cheraghi‐Sohi S , Jeffries M , Stevenson F , et al. The influence of personal communities on the self‐management of medication taking: a wider exploration of medicine work. Chronic Illn. 2015;11(2):77‐92. 10.1177/1742395314537841 24920009

[58] Powell C , Ismail H , Cleverley R , et al. Patients as qualitative data analysts: developing a method for a process evaluation of the “Improving the Safety and Continuity of Medicines management at care Transitions” (ISCOMAT) cluster randomised control trial. Health Expect. 2021;24(4):1254‐1262. 10.1111/hex.13257

[59] Mughal F , Khunti K , Mallen C . The impact of COVID‐19 on primary care: insights from the National Health Service (NHS) and future recommendations. J Fam Med Prim Care. 2021;10(12):4345. 10.4103/jfmpc.jfmpc_756_21 PMC7612361 35165652

[60] Khalil‐Khan A , Khan MA . The impact of COVID‐19 on primary care: a scoping review. Cureus. 2023;15(1):e33241. 10.7759/cureus.33241 36618499 PMC9815485

[61] Fraser C , Fisher R . How has the COVID‐19 pandemic impacted primary care? The Health Foundation. May 27, 2021. Accessed August 30, 2023. https://www.health.org.uk/news-and-comment/charts-and-infographics/how-has-the-covid-19-pandemic-impacted-primary-care

[62] Chudasama YV , Gillies CL , Zaccardi F , et al. Impact of COVID‐19 on routine care for chronic diseases: a global survey of views from healthcare professionals. Diabetes Metab Syndr. 2020;14(5):965‐967. 10.1016/j.dsx.2020.06.042 32604016 PMC7308780

[63] Regional ethnic diversity . Ethnicity facts and figures. Government data about the UK's different ethnic groups. December 22, 2022. Accessed August 30, 2023. https://www.ethnicity-facts-figures.service.gov.uk/uk-population-by-ethnicity/national-and-regional-populations/regional-ethnic-diversity/latest

[64] The Law Society . A guide to race and ethnicity terminology and language. The Law Society. June 27, 2023. Accessed August 30, 2023. https://www.lawsociety.org.uk/topics/ethnic-minority-lawyers/a-guide-to-race-and-ethnicity-terminology-and-language

[65] Norman C , Wildman JM , Sowden S . COVID‐19 at the deep end: a qualitative interview study of primary care staff working in the most deprived areas of England during the COVID‐19 pandemic. Int J Environ Res Public Health. 2021;18(16):8689. 10.3390/ijerph18168689 34444437 PMC8393368

