# Supplementary material for: ‘It's a job to be done’. Managing polypharmacy at home: A qualitative interview study exploring the experiences of older people living with frailty
Source: Health Expect. 2024 Jan 10;27(1):e13952. doi: 10.1111/hex.13952 (PMC10777610; doi:10.1111/hex.13952)
Supplement: Supplementary file 1 — Supporting information. [file HEX-27-e13952-s002.docx]

**Appendix 1**

**Patient interview schedule**

**Understanding and enhancing medicines management resilience in older people living at home**

Introduction and thanks, permission to record, explanation of what will happened during the interview, recap on purpose of the study, explain how to take a break or stop the interview.

**About you**

Age, ethnicity, number of medicines.

**First of all, please can you tell me about the medicines you take, for example what they are for and how often you take them?**

Prompts: What do take them for? How do you find out about them? How do you feel about them? Where do you find information about them? How often do your medicines or doses change? How do you manage those changes? Who would you ask if you have a question about them? Have you asked any questions about them? Who do you talk to about them?

**Who, if anyone, helps or supports you in managing your medicines?**

Prompts: What sorts of things do they help you with? How do you decide who does which task? How much does this help you to remember information about your medicines, or help you manage them? How do you get help with your medicines? How do you know that you need help? What would you do if you think you might be struggling to manage your medicines? How would you know if you were struggling to manage them? Who would you tell if you were struggling?

**Please talk me through how manage your medicines day-to-day.**

Prompts: How do you order you medicines? How do you collect them? Or does someone drop them off? And how do manage them at home? Do you keep them in a particular place? What do you do with the medicines you no longer need? What do you do if you have questions about them? What do you do if the medicines you get aren’t what you are expecting? Do you check the medicines when you get them? What do you do? Have you had any problems in the past with your medicines? What did you do? How did you decide what to do? Do you check your repeat prescriptions? Why do you do that? Do you check your prescriptions after your medicines have been changed?

**How do you manage your medicines?**

How about lists, checklists, calendars, diaries, containers, reminders? What kind of changes do you make when you have new or changed medicines? How do you check how you are managing your medicines?

**Have you ever had any worries or other difficulties with your medicines?**

Can you tell me a little bit more about that? What did you do? Who, if anyone, did you go to for help? Which healthcare people, if any, did you contact? What about when your medicines have changed? How well did you understand the changes? Did you ask for help? Who did you ask?

**How do you keep an eye on your own health?**

What do you do if you are feeling unwell? Whose help would you ask for, if anyone’s? What would you do if you are not feeling very well? Would you keep an eye on how you are feeling after your medicines have been changed?

Debrief and thanks
