# Supplementary material for: ‘It's a job to be done’. Managing polypharmacy at home: A qualitative interview study exploring the experiences of older people living with frailty
Source: Health Expect. 2024 Jan 10;27(1):e13952. doi: 10.1111/hex.13952 (PMC10777610; doi:10.1111/hex.13952)
Supplement: Supplementary file 2 — Supporting information. [file HEX-27-e13952-s001.docx]

# **Appendix 2**

# **Reflexivity in Thematic Analysis**

To be consistent with the reflexive approach I will write these reflections in first person.[^61^](https://paperpile.com/c/bqNyln/UMgKy) I am a white non-British social researcher who moved to the UK nine years ago. I do not take medicines regularly and I have very limited experiences of supporting someone taking 5 or more medicines. My knowledge of the UK healthcare system as a patient is very limited. My orientation was experiential. I wanted to try and understand how medicines management was experienced by older patients, but I was very aware that my personal and professional experiences of medicines management at the start of this study were very limited. Our research team decided to approach data familiarisation working in group and facilitating involvement of our patients and family advisory group.

As the first tranche of interviews transcripts became available (10 interviews) we shared them across our research team. The team included pharmacists, social researchers and an older patient with lived experience of polypharmacy. Group discussions were held about the ontological and epistemological assumptions each of us made when looking at the data. We also shared the ways in which the data resonated with our personal and professional experience as clinicians, researchers and/or relatives of someone taking multiple medicines in older age.

To facilitate contribution from patients and family members in the advisory group (2 older patients and 3 family members), CP and I set up an introductory workshop on qualitative research and thematic analysis. In the second part of the workshop the group familiarised with one interview transcript and made notes on it. They also had the opportunity to familiarise and annotate additional transcripts if they wanted to do. After that, CP and I run a group discussion where everyone shared what attracted their attention in the transcripts they looked at and why.

Listening to what captured staff, patients and family members' attention while reading the transcripts “helped me to switch on my senses” and opened up my way of looking at the data. It also felt right and consistent with the study aim (co-design an intervention to support frail older patients with polypharmacy) to have structured input on the meaning of the data from the groups that are going to benefit from the final output.

After team discussion, it was decided^^[[1]](#footnote-0)^^ that I (GPr) would code all the data, after considering the notes and codes’ suggestions made by researchers, patients and family members. Our aim was not to try and standardise the way we were coding but to build the most comprehensive and nuanced interpretation of the data, including multiple perspectives (patient’s view, family supporter view, social researcher view, clinical researcher view).

After completing familiarisation and coding of all the 32 interviews conducted with patients, I reviewed all the codes and the related data extraction to make sure they were meaningful in relation to the research questions. The approach I followed was predominantly inductive, as data were open-coded. Resilient healthcare worked as a sensitising concept to select what part of the data provided answers to the research questions.

Some codes covering different aspects of the same idea were merged together and repetitions were resolved. When I was sufficiently happy with the codes identified and revised, I started clustering them. Working with BF, we formed 3 provisional themes, each with 3 provisional subthemes.

I then started working on reporting the findings, writing up the definition for each theme core idea. While defining the themes I realised that some were not fully developed. In some themes the core idea was simply not strong and meaningful enough to pass the iteration. Going back and forward between the raw data, the codes, and the clusters BF and I shaped new patterns which were able to tell a more analytically developed story about the data.

1. [Trainor LR, Bundon A. Developing the craft: reflexive accounts of doing reflexive thematic analysis. *Qualitative Research in Sport, Exercise and Health*. 2021;13(5):705-726. doi:](http://paperpile.com/b/bqNyln/UMgKy)[10.1080/2159676X.2020.1840423](http://dx.doi.org/10.1080/2159676X.2020.1840423) [↑](#footnote-ref-0)
