# Supplementary material for: ‘It's a job to be done’. Managing polypharmacy at home: A qualitative interview study exploring the experiences of older people living with frailty
Source: Health Expect. 2024 Jan 10;27(1):e13952. doi: 10.1111/hex.13952 (PMC10777610; doi:10.1111/hex.13952)
Supplement: Supplementary file 3 — Supporting information. [file HEX-27-e13952-s003.docx]

Appendix 3 Participant characteristics

| **Patient ID** | **Ethnicity** | **Medicines** | **Gender** | **Age band** | **Interview type** |
| --- | --- | --- | --- | --- | --- |
| PP1 | White British | 5 | M | 80-84 | Face to face |
| PP2 | White British | 5 | F | 65-69 | Online |
| PP3 | White British | 5 | M | 65-69 | Phone |
| PP4 | White British | 9 | F | 85+ | Phone |
| PP5 | White British | 6 | F | 70-74 | Face to face |
| PP6 | White British | 14 | M | 75-79 | Face to face |
| PP7 | White British | 6 | F | 75-79 | Face to face |
| PP8 | White British | 6 | M | 80-84 | Online |
| PP9 | White British | 6 | F | 80-84 | Face to face |
| PP10 | White British | 6 | F | 75-79 | Online |
| PP11 | Asian British (Pakistani) | 11 | M | 65-69 | Face to face |
| PP12 | White British | 15 | M | 75-79 | Face to face |
| PP13 | White British | 9 | M | 65-69 | Online |
| PP14 | White British | 8 | F | 80-84 | Face to face |
| PP15 | White British | 5 | M | 80-84 | Online |
| PP16 | White British | 8 | F | 70-74 | Face to face |
| PP17 | White British | 9 | M | 80-84 | Online |
| PP18 | White British | 9 | F | 80-84 | Phone |
| PP19 | White British | 6 | M | 85+ | Phone |
| **Patient ID** | **Ethnicity** | **Medicines** | **Gender** | **Age band** | **Interview type** |
| PP20 | Other Ethnic Groups (South American) | 8 | M | 75-79 | Online |
| PP21 | White British | 6 | M | 75-79 | Phone |
| PP22 | White British | 8 | F | 80-84 | Phone |
| PP23 | White British | 13 | M | 80-84 | Online |
| PP24 | White British | 7 | M | 85+ | Phone |
| PP25 | White British | 6 | F | 80-84 | Phone |
| PP26 | White British | 7 | M | 70-74 | Online |
| PP27 | White British | 5 | M | 70-74 | Phone |
| PP28 | British white | 6 | F | 65-69 | Phone |
| PP29 | British white | 8 | M | 80-84 | Face to face |
| PP30 | British white | 8 | F | 80-84 | Face to face |
| PP31 | British white | 6 | F | 70-74 | Online |
| PP32 | British white | 7 | F | 80-84 | Phone |
